# A Social Justice Field Education Model for Addressing Isolation and Other Social Determinants of Behavioral Health

**DOI:** 10.1093/geroni/igaa057.2311

**Published:** 2020-12-16

**Authors:** Karen Bullock, Kim Stansbury, David Fitzpatrick

**Affiliations:** NC State University, Raleigh, North Carolina, United States

## Abstract

Behavioral health and substance use disorders are significant problems among older adults who are experiencing homelessness. This presentation describes the components of a social work field education model for preparing practitioners to work effectively in community-based agencies using the Hartford Practicum Partnership in Aging Educational (HPPAE) model. A planned and systematic transition from one field setting to another with the goal of giving students a range of practice experiences with various service delivery systems to address social isolation and other social determinants of health. This exploration of demographic and social action factors that may impede or foster success among community-dwelling older adults offer insight and guidance for constructing a conceptual and theoretical model a social justice framework in geriatric social work practice and research.

